# Terahertz spectrum-based refractive index sensor for brain lesion detection using photonic crystal fibers

**DOI:** 10.1371/journal.pone.0320355

**Published:** 2025-03-26

**Authors:** Md. Naimur Rahman Naim, Jabin Tasnin Upoma, A. H. M. Iftekharul Ferdous, Kayab Khandakar, Md. Golam Sadeque, Md. Sohel Rana

**Affiliations:** Department of Electrical and Electronic Engineering, Pabna University of Science and Technology, Pabna, Bangladesh; DIT University, INDIA

## Abstract

Numerous types of brain lesions, cancers, and tumors are still thought to be lethal. An accurate photonic-based biological sensor that is capable of differentiate between normal and pathological brain tissues is presented in this work. Here, we use the defective two-dimensional photonic crystal to theoretically investigate the brain lesion detection. By monitoring the red shift of the wavelength of resonance that occurs as the layer of cerebral lesions’ RI (Refractive Index) shifts from 1.38 to 1.4591, the device’s execution is confirmed. The outcomes of the simulation using the proposed detector architecture yield very high relative sensitivity values of 98.54%, 98.84%, 99.049% and 97.075%; low EML values of 0.00359 cm^-1^, 0.00323 cm^-1^, 0.00297 cm^-1^, 0.00533 cm^-1^ for Low Grade Glioma, Glio-blastoma, Lymphoma and Normal brain cell, respectively, at a 2.2 THz frequency regime. Furthermore, the simulation results for each of the cancer cases stated point to a remarkably minimal CL (Confinement Loss) of 6.315 ×  10^-15^ dB/cm. Through its ability to sense even minute fluctuations in RI, our planned sensor facilitates the identification of tumor at low concentrations. Because of its rapid reflexes, it can monitor the quantities of Low Grade Glioma, Glio-blastoma, Lymphoma and Normal brain cell instantly, which is essential to act rapidly in dangerous situations. In general, the efficacy, accuracy, and versatility of newly developed PCF sensors make them valuable tools for the reliable detection of Low Grade Glioma, Glio-blastoma, Lymphoma and Normal brain cell in a range of contexts, enhancing safety and surveillance protocols.

## 1. Introduction

Although the human body possesses a strong immune system that can fight off any illness, certain lesions can lead to more serious illnesses. One of these is brain cancer, for which early discovery is helpful in terms of treatment. Numerous recently developed diagnostic methods for the identification of cancer cells are available at the micro and Nano scales. Given that the cells’ refractive index (RI) indicates whether they are aberrant or normal, it is also an indicator of their physical states. As a result, sensors based on optical waveguides have great promise for the identification of malignant cells. Several research groups have successfully proved the identification of cancerous brain cells by 1D photonic crystal transmission spectrum analysis [1]. The primary purpose of optical fiber innovation was for telecommunications. On the other hand, directed wave technological advances has expanded for uses in detection partly because of optical fibers, thanks to advancements in manufacturing technology. The advantages of optical fibers for biochemical sensing applications have been shown by the creation of PCFs and their numerous possible uses [2–4]. Many researchers are interested in the PCF because of its exceptional photonic features, which include unending single mode [5], low loss [6], strong birefringence [7], effective mode field size [8], dispersion [9], and tremendous nonlinearity. PCF devices hold great potential in the fields of fiber medical imaging [10], sensing [11], telecommunication [12], spectroscopy [13], and environmental applications [14]. They can also be used in pharmaceutical drug testing [15]. Shakya et al. Propose using a High-Reflective [HR] coating on the top and bottom flat surfaces of a disc to support a vertical FP mode, which resonantly improves pump acceptance [16]. PCF SPR biosensors use their ability to detect minute differences in refractive index (RI) values, which is critical for distinguishing between healthy and abnormal cells. Historically, the geometric designs of PCF SPR biosensors were divided into three categories: internal metal deposition (IMD), external metal deposition (EMD), and D-shaped geometry [17–19]. All these methodologies possess advantages and shortcomings, like PCFSPR sensors based on IMD techniques have a coating of plasmonic material on the internal air hole within the PCF. This methodology is highly complicated from a fabrication perspective. In the D-shaped PCF one surface of the fiber is polished to provide a flat surface to give the fiber a D-shaped structure. This methodology is again challenging from a fabrication perspective. Finally, the fiber structures based on the external metal deposition (EMD) approach are less complicated than IMD and D-shaped PCF structure [20]. Traditional plasmonic materials such as aluminium (Al), copper (Cu), gold (Au), silver (Ag), and graphene have been widely used to produce the SPR effect. The spectroscopic analysis is carried out with the use of research equipment and installations. In order to build genuine chemometric techniques, spectroscopy-based sensor setups also heavily rely on processors [21].

Surface Plasmon resonance (SPR) is a collective electronic oscillation phenomenon that can be divided into two types based on their respective excitation methods: Surface Plasmon Polarisation (SPP) and Localised Surface Plasmon Resonance (LSPR) [22,23]. SPP can overcome the diffraction limit and improve light manipulation at the subwavelength scale. It is typically produced by shining plane-polarized light onto a continuous metal surface. As a result, it is a promising candidate for the next generation of ultra-miniature integrated photonic circuits and highly sensitive biosensors for data processing. SPR sensors are widely used in biosensing applications due to their high sensitivity and label-free detection abilities. SPR sensors rely on an optical phenomenon at the interface of a thin metal sheet and a dielectric medium [24]. Although traditional single-metal biosensors based on prisms are less sensitive, researchers are constantly trying to improve their performance parameters [25]. In the context of SPR-based sensors, a surface Plasmon wave (SPW) is produced when an electron density wave travels along an interface between a material with a positive (dielectric) constant and a negative (metal) constant; light incident on the SPW stimulates its resonant frequency. One advantage of the SPR technique is the ability to track in real time the interactions of specific analyses with biomolecules, such as enzymes or antibodies that have been immobilised on a sensing surface in real time [26]. While THz communications hold great potential for ultra-broadband wireless systems, path attenuation and possible application situations necessitate sophisticated hardware and software solutions [27]. With its enormous effective mode area, a large amount of creative freedom, and single-mode guidance, PCF is a unique kind of optical fibre. PCF has been utilized more and more for substance, gas, and biological sensing applications in the THz and IR sectors throughout the past ten years [28,29]. Much curiosity about research is currently shown in the terahertz (THz) electromagnetic range. It crosses across to the infrared and microwave bands, with a frequency between 0.1 THz and 10 THz. Among these two radiation types, this range substantially narrows the frequency difference. THz radiation speeds up laser communication, in contrast to microwave radiation, whose lower frequency allows for only a restricted data transfer rate [30]. Its applications can be compared to those of other well-known technologies such as nondestructive method of analysis [31], inline measurements in plastic extrusion processes [32], analyzing plastic components and finding errors [33], high accuracy and selectivity of gas phase sample measurements [34], larger bandwidth [35], tissue detection [36], non-destructive testing (NDT) [37] and detect materials [38]. Terahertz waves have potential in medicine because they carry detailed information and are safe to use, but accurately identifying tumors with them is still tricky due to limitations in resolution and interpreting the data, so scientists from different fields need to work together to make it work in real medical settings[39].

It has not been established how to use complicated refractive index values for THz imaging of gliomas in fresh tissues to differentiate between tumor and normal tissues [40]. For the purpose of identifying malignant tissue, The wide-band range of frequencies of pulsed terahertz frequency is 0.1 THz to 4 THz [41]. THz imaging allowed for the distinction of the various TBI degrees. Every year, 10 million individuals die and are admitted to hospitals due to Traumatic Brain Injury (TBI), and 57 million people worldwide have had TBI, with between 75 and 85 percent of cases being classified as mild [42]. Brain tumors as well as other parts of the neurological apparatus (CNS) represent 1.7% of all cancer cases globally, this being the 17th most prevalent kind of cancer overall (not including non-melanoma skin cancer) [43]. Non-malignant tumors, like meningiomas, now surpass malignant tumors in prevalence, comprising 71.7% of all brain and CNS cancers. Glioblastomas constitute 50.1% of malignant tumors, while meningiomas account for 55.4% of tumors not malignant [44]. The National Brain Tumor Foundation (NBTF) states that brain tumors are becoming more common causes of death in developed nations [45]. The greatest number of types of brain cancer affecting kids is known as medulloblastoma, primary brain and CNS malignancies are among the most prevalent material tumors among children [46]. In 2022, an estimated 40,594 kids and teenagers in the range of 0 and 19 had been identified as having central brain tumors and additional tumors of the neurological system [47]. Among various photonic-based sensing methods, PC-based sensors have recently been seen as a potentially useful approach [48]. Over the past ten years, PC has been utilized to deliver precise, gas sensor [49], refractive index (RI) sensors [50] and biochemical sensors [51], pH sensors [52], strain sensor [53] and so on. Both cancerous and normal cells have penetrated the PC cavity. Numerous optical resonant structures in silicon have been proposed, including two-dimensional (2D) and one-dimensional (1D) photonic crystal (PC) cavities [54]. The position of a transmission resonant peak inside the PBG that results from a defect layer submerged in the 1D-PC depends on the defect layer’s refractive index [55]. For the purpose of assessing the RI of CNS tissues, maintaining tissue viability may be crucial [27]. 2D-PC capable of identifying cancer cells [56]. A variety of films have been applied to the sensor’s surface to improve performance. Researchers have recently become interested in two-dimensional (2D) materials, a novel class of materials, because of their superior photoelectric properties. According to the research, transition metal dichalcogenides (TMDCs) are comparatively two-dimensional materials whose exceptional qualities have prompted research into their potential application in SPR sensors [57]. The potential of two-dimensional (2D) materials, like TMDCs, to enhance SPR sensor performance has recently attracted a lot of attention due to their distinct electrical and optical characteristics [58]. The crucial parameter is sensitivity, which ought to be high. The addition of the two-dimensional (2D) material to the conventional sensor can increase the sensitivity of conventional biosensors [59]. Advances in technology have led to the use of 2D materials in sensing applications. SPR-based sensors are increasingly using the most popular 2D materials [60], like graphene, MXene, and black phosphorus, because of their optoelectrical properties [61]. Researchers have recently focused on MXene, a two-dimensional material, due to its mechanical and electrical properties [62].

The main goal of creating a photonic-based biological sensor with terahertz frequency and photonic crystal fibers is to identify brain lesions with high sensitivity and accuracy. By taking advantage of the differences in refractive index in brain tissues, the sensor uses the terahertz frequency to distinguish between healthy and infectious tissues, thereby offering a non-invasive, effective, and real-time diagnostic tool with improved precision, overcoming the shortcomings of traditional imaging approaches in identifying abnormalities in the brain. This study proposes and statistically evaluates a novel ten symmetrical air holes PCF with circular core for THz-based tumor identification. We have determined that the recommended sensor’s function frequency should be 2.2 THz since this frequency achieves maximum the sensor’s RS. Our proposed sensor performs better at identifying these types of cancer cells than previous study that is identified within the scientific review because it has high relative sensitivities that are comparable to Low Grade Glioma, Glio-blastoma, Lymphoma and Normal brain cell at 2.2 THz (98.54%, 98.84%, 99.049% and 97.075%). Accordingly, at 2.2 THz, the NA values for Low Grade Glioma, Glio-blastoma, Lymphoma and Normal brain cell with extremely little loss are 0.2641, 0.2646, 0.2651 and 0.2622, correspondingly.

## 2. Methodology

**Fig 1:**
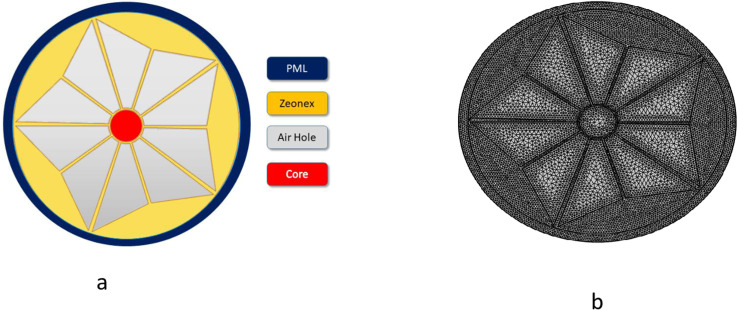
(a) Represent the innovative Photonic Crystals Fiber cross-segment. (b) At present designed PCF mesh structure.

COMSOL Multiphysics The expected PCF detector was efficiently designed and analyzed with the use of simulation software. In this research, we aim to detect changes in refractive index (RI) using 2D photonic crystals as a standard method for distinguishing between normal and cancerous brain tissues during medical procedure. The suggested 2D photonic crystal sensor uses terahertz (THz) spectroscopy to analyze changes in the refractive index in order to distinguish between healthy and diseased brain regions. The sensor uses photonic crystal fibers (PCFs) to direct THz waves through the tissue sample. Because diseased tissues have different biochemical compositions, they show noticeable alterations in refractive index. By altering the transmitted spectrum, these shifts make it possible to precisely identify and describe brain lesions. Because of the design’s great sensitivity and selectivity, it is possible to distinguish between healthy and sick tissues using their distinct optical characteristics. The inclusion of Zeonex, known for its transparency and low optical loss, further boosts performance, while the unique structure with perfectly matched layers reduces back-reflection and improves efficiency. The simple manufacturing process ensures cost-effective production, making this sensor an effective tool for brain lesion detection. The air channels composed of flower petals comprise the unique coating region of the constructed detector. These cores have the benefit of being easily deceitful. The circular core maximizes the sensing potential and facilitates the convenient placement of a larger number of objects within it. Zeonex is one of our main compounds and is used as the foundation. The PCF sensor, developed using Zeonex, offers high transparency, low absorption loss, and biocompatibility, making it ideal for detecting refractive index variations in brain lesions. Its round core with flower-petal-shaped air channels enhances light confinement and simplifies fabrication. A mesh design and Perfectly Matched Layer improve beam guidance and efficiency, making it a cost-effective and sensitive tool for medical diagnostics. It is perfect for biological sensors because it has low optical loss, great clarity, and biocompatibility. However, compared to substances like polymethyl methacrylate (PMMA) or silica, this material has significant drawbacks, such as less flexibility in altering characteristics and production difficulties. Zeonex accounts for the value of RI = 1.53 [63]. On the clad layer, ten composite air intakes are created overall. In this case, RI = 1 air is being examined. Nonetheless, radiation therapy has shaped normal brain cells, glioblastoma, low-grade glioma, and lymphoma inside a circular core. Here, the stated lesions with RI values of 1.432, 1.4470, 1.4591, and 1.38 are taken into account [64]. When employing terahertz spectrum-based refractive index sensors to identify brain lesions, tracking the red shift in resonance frequency is essential. The great sensitivity of photonic crystal fibers (PCFs) to shifts in refractive index brought on by diseased tissues is part of their design. A higher refractive index is shown by a red shift in the resonance frequency, which helps to differentiate healthy tissue from lesioned parts. This technique makes use of the accuracy of the terahertz frequency and the special optical characteristics of PCFs to diagnose brain lesions accurately and non-invasively. Refractive index variation from 1.38 to 1.4591 demonstrates the detector’s sensitivity to cell structure changes, which is essential for identifying brain lesions. Brain lesions cause changes in tissue characteristics including cell density and water content, which are associated to this shift in the refractive index. By calibrating the sensor’s response for particular refractive index ranges, analytes can be distinguished from one another, and complementing methods like as scanning or spectral analysis can be employed to differentiate between brain tissues and other substances. By offering high sensitivity to variations in the optical characteristics of the tissue, the sensor’s capacity to identify even the smallest refractive index (RI) fluctuations increases its usefulness in tumor diagnosis. Because of their changed cellular architecture and increasing tissue density, tumors frequently result in minor fluctuations in the RI. Early tumor diagnosis and enhanced diagnostic accuracy are made possible by the high precision detection of these minute variations utilizing a refractive index sensor based on the terahertz band and photonic crystal fibers. This technique provides real-time, non-invasive brain lesion monitoring.The area of the sectional view for the recommended Photonic Crystals Fiber is illustrated in Fig 1(a) in this instance.

The figure, known as Fig 1a, offers a thorough illustration of a circularly cored construction.

This circular core’s radius is explicitly indicated as C =  0.768P. There is a covering layer in the framework as well. Ten sectors are created with a fixed outer radius of R1 = 0.8352 * P and a sector angle of 30 degrees. Five sectors, each with a fixed sector angle of 60 degrees and a radius of R2 =  3.84 * P, have been designed through rotation. Five additional sectors, each with a radius of R3 =  5.28 *  P and a sector angle of 60 degrees, have been created, with a rotation 36 degrees greater than the previous ones. By eliminating the inner arms of the sectors and joining them with line segments, the connection is made correctly, as illustrated in fig-1(a). The Perfectly Matched Layer (PML) has two distinct inner and outer radii: L1 =  5.376P and L2 =  5.76P, respectively. These designations are made simultaneously. The range of the variable “P” in this context is 150-250 µm. The Perfectly Matched Layer is a special level which is placed in the greatest portion of the fiber with purpose. Its main purpose is to collect any radiation that may seep out of the central portion on the exterior of the coating topography. This greatly lowers back-reflection and raises the fiber’s total efficiency. Because it has been correctly matched to the remainder of the fiber framework, it will function at its best because it is made to blend in effortlessly. A procedure of experimentation and error is used to determine the variables in problem. The suggested sensor has some advantageous characteristics, which is why this approach is used. The PCF sensor, designed using COMSOL Multiphysics, detects minute refractive index (RI) changes with high sensitivity, distinguishing between normal brain tissues and lesions. Its flower-petal air channel structure enhances light confinement, while Zeonex, with RI =  1.53, ensures transparency and low loss. A circular core and cladding geometry maximize sensing potential while reducing complexity, supported by a mesh design for precision. The Perfectly Matched Layer minimizes back reflection, enabling accurate RI detection from 1.38 to 1.53, making it effective for brain lesion diagnosis. A low loss profile, increased sensitivity, and the capacity to guarantee enhanced light confinement are a few of these. The holes in the air found in the coating and core exhibit a range of forms. The general effectiveness and functioning of the detector are enhanced by this shape diversity. The fact that this design helps to simplify the process of manufacturing is one of its many noteworthy benefits. This is mainly due to the holes in the fiber cores and cladding are determined by a single constant (P). The layout streamlines the production method and increases its efficiency and cost-effectiveness by reducing the amount of factors. This method makes it simpler and less costly to create a detector while also guaranteeing that it will continue to function at a high level.

The term “mesh” in PCF sensor technology refers to the intricate structure of the carbohydrates, which are expertly engineered to generate pleasing optical as well as identifying properties spanning a range of applications. The PCF’s mesh design has a significant impact on its transparency, which includes beam guidance and a link to external parts that permits observation. It is feasible to meticulously adjust the ventilation chamber organization of that mesh to modify its noise properties, variation, and susceptibility to various components of the fiber. Mesh offers: A total of 5971 elements and 52 polygon parts; 910 boundary units alongside 5971 elements overall; lowest feature grade 33971. Where the mesh architecture for the added Photonic Crystals Fiber is displayed in Fig 1(b).

**Fig 2:**
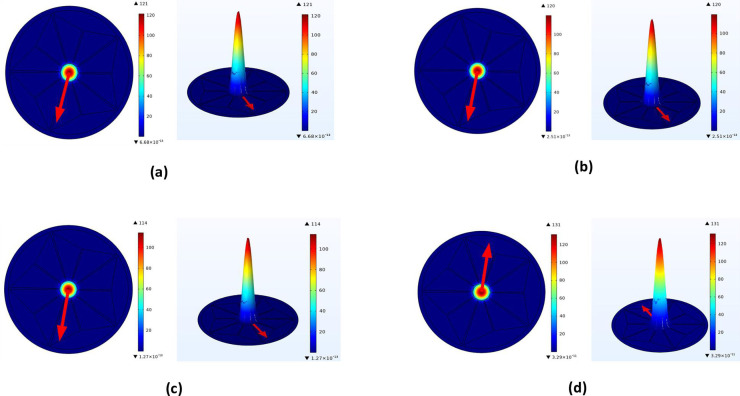
(a) Apparent dispersion of Low Grade Glioma (a) Power (b) Density. (b) Apparent dispersion of Low Glio-blastoma (a) Power (b) Density. (c) Apparent dispersion of Lymphoma (a) Power (b) Density. (d) Apparent dispersion of Normal cell (a) Power (b) Density.

In general, density dispersion explains how a material scatters around a Photonic Crystals Fiber display which is illustrated in Fig 2. It makes clear how a substance’s elasticity differs according to it center, its peripheral, as well as different functional fibre sections. Granular dispersion must be understood to construct Photonic Crystals Fiber with certain responsibility, orientated properties, or reactions to outside influences in applications for communication and observation.

## 3. Results and Analyses

The design, simulation, and analysis of the brain lesion-detecting PCF sensor in COMSOL Multiphysics 6.1, using the finite element method (FEM), are crucial for optimizing performance. The geometry, featuring a circular core and flower-petal-shaped air channels, is simulated to achieve maximum light confinement and sensitivity. FEM allows for the accurate modeling of complex structures, including Perfectly Matched Layers (PML) to reduce back-reflection. The software facilitates the iteration of key parameters like refractive index, dimensions, and material properties, ensuring high performance and cost efficiency in medical diagnostics. This approach simplifies manufacturing and enhances the sensor’s optical and sensing properties [65]. The PCF sensor design in COMSOL Multiphysics utilized structural mechanics and wave optics modules to optimize core geometry and air hole arrangements for enhanced light confinement and sensing. The Perfectly Matched Layer reduced back-reflection and increased fiber efficiency, while meshing ensured accurate modeling. Material properties like refractive index and transparency were included for realistic simulations, and parametric sweeps optimized variables for low loss and high sensitivity. This approach demonstrated a cost-effective and efficient PCF sensor for terahertz spectrum-based brain lesion detection. The photonic-based sensor outperforms conventional imaging techniques like MRI and CT scans by detecting subtle tissue changes through terahertz spectrum refractive index variations. Designed with COMSOL Multiphysics, it features a circular core and optimized flower-petal air channel arrangement for enhanced sensitivity and low loss. Using Zeonex, the sensor ensures high transparency, low optical loss, and biocompatibility, overcoming limitations of traditional materials like PMMA or silica. The Perfectly Matched Layer minimizes back-reflection, boosting accuracy, making this sensor a highly sensitive and non-invasive tool for brain lesion detection.

The Maxwell’s formula is solved numerically throughout the entire process using the FEM approach [66].The characteristics of the PCF being utilised as the sensor must first be understood in order to compute the relative sensitivity response. A PCF’s proposed detecting abilities were indicated by its RS response. A PCF’s relative sensitivity is the degree of its optical characteristics adapt to shifting environmental conditions, like variations in pressure or temperature. A photonic crystal fibre’s level of sensitivity is in comparison relating to other materials is dependent upon a number of various elements, such as the fiber architecture, the spectrum of the light as well as the properties of the environment around it. The maximum relative sensitivity of the sensor is estimated on the basis of the difference between the effective refractive indices of the core and the cladding of the PCF sensor, which are dependent on its structural and material composition. The proposed design assumes a Zeonex-based circular core with a refractive index of 1.53, which provides the best light confinement and interaction with target samples consisting of normal brain cells, glioblastoma, low-grade glioma, and lymphoma with refractive index values of 1.38, 1.4591, 1.4470, and 1.432, respectively. The unique configuration of air holes in the cladding, set as flower petals, enhances sensitivity by ensuring more precise guidance of light and hence minimum losses. It integrates a perfectly matched layer to minimize the back-reflection for better efficiency. Large density elements in the mesh structure are chosen to get optimum beam guidance and response of variation in an external refractive index. All these factors enhance the sensitivity of the sensor, including the material properties of high transparency and biocompatibility of Zeonex, the structural features of a circular core and air channels, and design optimizations like the PML. This allows distinguishing effectively between glioma, glioblastoma, lymphoma, and normal brain tissues, making the sensor a powerful tool in the detection of brain lesions. Photonic Crystal Fibre consistently shows a greater sensitivity in comparison to traditional optical fibres due of its huge surface scale and compact core. Beer-Lambert’s law states that a variation in relative sensitivity that is linear results from changes in the relationship between radiation and matter. Use the formula that follows to determine any sensor’s corresponding location [67].


$$r=\frac{n_{r}}{n_{eff}}\times p\%$$ (1)

where denotes the power fraction p, which may be calculated applying the following calculation, and *n*_*r*_ is the actual percentage of the Reflecting Index of the detecting analyte in addition to the *ne*_*ff*_, or guided mode index of refraction, using the following formula [67].


$$p=\frac{\int_{sample}^{}R_{e}\left(E_{x}H_{y}-E_{y}H_{x}\right)dxdy}{\int_{total}^{}R_{e}\left(E_{x}H_{y}-E_{y}H_{x}\right)dxdy}$$ (2)

**Fig 3:**
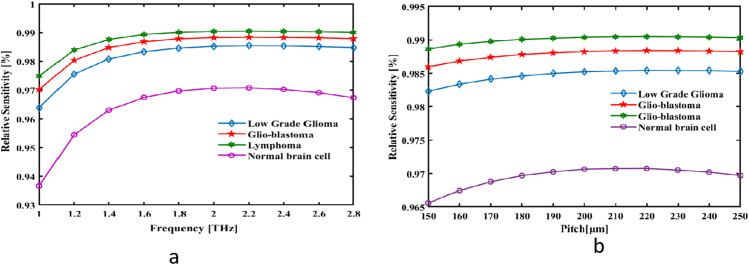
The suggested detecting analyte’s RS is represented as a function of (a) pitch at f = ** 2.2 THz and (b) fixed pitch signal frequency at 220 µm.**

Where, H_x_ and H_y_ show the magnetic field in both the x and y axis and E_x_ and $E_{y}$ indicate the elements of the electric field pointing in the x and y instructions. A sensitivity graph accounting for variations in frequency is displayed in Fig 3(a). While increasing the frequency in the terahertz range, the coupling of the electromagnetic waves with refractive index variations in the tissue samples becomes stronger, which can identify very minute structural changes in brain lesions. Design parameters include pitch (P) and an intricate mesh structure that optimizes air channels in the cladding and core for better light guidance with minimum loss. The round core maximizes the interaction area, whose radius is linearly proportional to the pitch for the enhancement of the sensor detecting subtle refractive index changes. The Perfectly Matched Layer further eliminates back-reflection for efficient operation at higher sensitivity. Usually, tumors cause a slight variation in RI due to their changed cellular architecture and increased density of tissues. This makes accurate detection of RI highly essential for the early diagnosis by the sensor. The sectoral layers and rotational geometry resemble the waveguide-like geometry, resulting in an effectively confined light that enhances sensitivity and diminishes losses. The rich interplay of frequency-dependent light-matter interaction, structural optimization, and low-loss design leads to high relative sensitivity of the sensor, thus enabling the real-time detection of brain lesions with high accuracy.An analysis of the recommended Photonic Crystal Fibre (PCF)-based Reflected Index (RI) computationally sensor has taken place spanning the 1.0–2.8 THz range. The detector can identify 98.54% of Low Grade Glioma, 98.84% of Glio-blastoma, 99.049% of Lymphoma, and 97.075% of Normal brain cell at the optimal pitch and frequency. It is evident that the optical response develops in frequency and peaks at 2.2 THz when plotted against a light confinement. The frequency of visual strength contact with analyte or minimal restraint rises and peaks at 2.2 THz, according to the plot. Fig 3(b) displays the pitch-variable sensitivity curve. The pitch parameter increases (from 150 to 250 µm), light confinement increases as well, reaching its maximum at 220 µm.

Regarding the proposed sensor, two noticeable types of difficulties are mentioned: EML and CL. These difficulties take place in all types of optical waveguides. EML is the most important THz waveguide directing characteristic. EML means Effective Material Loss. The EML (Effective Material Loss) values for lesion and normal brain cell conditions are important because they show how material absorption and scattering properties differ. When exposed to terahertz radiation, these variations aid in differentiating between tissue states that are normal and ones that are abnormal. EML alterations are correlated with tissue composition, making it possible to detect lesions and abnormalities in brain tissue with high sensitivity. This is essential for early diagnosis and therapy planning.

The EML indicates power loss caused by the solid layout of the fibre. The expression for EML is as follows [67]:


$$\alpha_{eff}=\frac{{\left(\frac{\varepsilon_{0}}{\mu_{0}}\right)}^{\frac{1}{2}}\int_{A_{max}}^{}n\alpha_{mat}{\left|E\right|}^{2}dA}{2\int_{ALL}^{}S_{z}dA}$$ (3)

**Fig 4:**
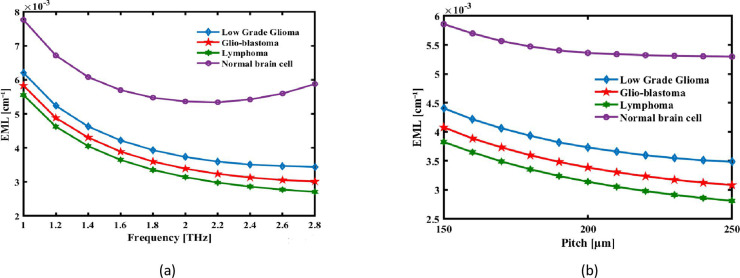
The EML of the recommended the detection is shown as an expression of (a) pitch at 2.2 THz and (b) signal frequency at a set pitch of 220 µm.

Considering that E is the modal electric field and αmat is the loss coefficient of the backdrop material. Fig 4(a) and 4(b) illustrates the modification of EML for different operational and structural conditions, correspondingly, for different THz frequencies and pitches at optimal settings.

This graph displays the declining aspects the decrease for longer cores. Zeonex loses power absorption capacity as more light flows across its longer core with reduced resistance. Thus, with an increase in frequency in the terahertz range, the electromagnetic waves experience better interaction with structured air channels and with a material featuring low absorption loss and highly transparent properties, such as Zeonex. A further increase in pitch (P) that is responsible for spacing of air holes in cladding enhances the capability of fiber to confine light within the core and minimizes energy escape to surrounding material. The design of PMLs contributes to the absorption of residual radiation effectively, reducing back-reflection and general losses. Moreover, the precise arrangement of the air holes and unique geometry of the circular core ensure an even distribution of the optical field that reduces hotspots generally contributing to material loss. Combination of the aforementioned structural and material properties has significantly low EML values, facilitating the efficient propagation of terahertz waves in order to give better performance related to detection by the sensor of refractive index variations related to brain lesions. As was earlier displayed, Greater frequency light is consistently confined restricted via the central. Consequently, when frequency increases, the suggested sensor’s EML falls, as seen in Fig 4(a). At 2.2 THz, the sensor fibre displays a remarkable low EML 0.00359 cm-1 of Low Grade Glioma, 0.00359 cm-1 of Glio-blastoma, 0.00297 cm-1 of Lymphoma, 0.00533 cm-1 of Normal brain cell.

Confinement loss is another significant issue to take into account when evaluating the detector’s performance. In a circuit, the air gaps act as a medium with dielectric properties. The spider-shaped air channels and hexagonal core in the PCF sensor enhance optical properties by improving mode confinement and reducing propagation loss. Spider-shaped air channels in the cladding increase light confinement and efficiency, while their specific orientation maximizes terahertz wave interaction with the medium for better sensitivity. The hexagonal core ensures a larger sensing area and efficient placement of sensing elements for accurate refractive index detection. A Perfectly Matched Layer absorbs stray radiation, further improving accuracy and reducing loss. Using Zeonex, with its high transparency, low absorption loss, and biocompatibility, ensures the sensor’s reliability in detecting brain lesions and other biological applications. Its relationship to the hypothetical part of the Effective Mode Index (EMI) is inverse. It is possible to compute leakage loss, or CL, precisely by adjusting the structural parameters. The following formula gives CL as [67]:


$$L_{c}=\frac{40\pi }{\ln\left(10\right)\lambda }img\left(n_{eff}\right)\times \frac{10^{6}dB}{m}$$ (4)

**Fig 5:**
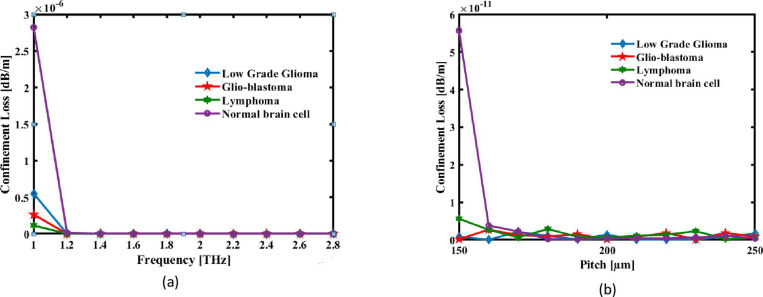
The CL of the suggested sensing analyte is plotted as an expression of (a) pitch at f =  **2.2 THz and (b) signal frequency at a set pitch 220µm.**

In this case, λ is the operational wavelength, and *Img, n*_*eff*_ denotes the portion of it that is imagined Effective Material Loss. The relationship between THz frequency and Confinement Loss is illustrated in Fig 5(a). Fig 5(b) illustrates how the value of Confinement Loss rapidly decreases for a range of pitches as the pitches in µm rise. At higher frequencies in the terahertz spectrum, the electromagnetic waves have shorter wavelengths and thus interact better with the structured air holes and material boundaries. Increased pitch (P, defining the spacing between the air holes in the cladding) gives a more effective photonic bandgap structure that allows the cladding to reflect and confine light within the core region, reducing electromagnetic energy leakage. The geometry and disposition of the circular core and cladding holes are accurately defined to reduce scattering losses and enhance mode confinement. Besides, stray radiation is captured by incorporating a perfectly matched layer, which effectively suppresses back-reflection and keeps high efficiency. These factors in totality collectively lead to smaller confinement loss. Smaller losses result in higher sensitivity; hence, sensors achieve better performances while detecting refractive index variations that correspond to lesions in the brain..It shows that when frequency and pitch raise, the Confinement Loss is falling. Under ideal circumstances, the CL of the sensing fibre is 6.315 ×  10^-15^ dB/cm. Figure illustrates the CL values for Low Grade Glioma, Glioblastoma, Lymphoma, and Normal Brain Cell at the optimal pitch of 220 µm and frequency at 2.2 THz, which are 4.8398 × 10^-13^ dB/m, 2.1415 × 10^-12^ dB/m, 1.0701 × 10^-13^ dB/m, and 1.3403 × 10^-12^ dB/m, respectively. The minimal confinement loss of 6.315 ×  10 ⁻ ¹⁵ dB/cm ensures that most of the light energy is confined within the sensor’s structure, minimizing signal loss. This enhances the sensor’s performance by improving sensitivity, maintaining high signal-to-noise ratios, and enabling accurate detection of subtle changes in tissue properties for brain lesion identification.

The Numerical Aperture is a quantity without units that falls between 0.1 and 0.5. A PCF’s Numerical Aperture is influenced by the variations in Reflected Index between the cladding and core materials in addition to the air hole or other imperfections that make up the photonic crystal lattice design. PCFs frequently have a higher NA than regular fibres, which makes them perfect for applications like high-power laser delivery, nonlinear optics, and sensing. The formula in may be applied to determine the type of light detector that is Numerical Aperture is ascertained by calculating in [68]:


$$NA=\frac{1}{\sqrt{1+\frac{\pi A_{eff}f^{2}}{c^{2}}}}\approx \frac{1}{\sqrt{1+\frac{\pi A_{eff}}{\lambda^{2}}}}$$ (5)

where λ indicates the response wavelength and Aeff the EA of the detecting element.

**Fig 6:**
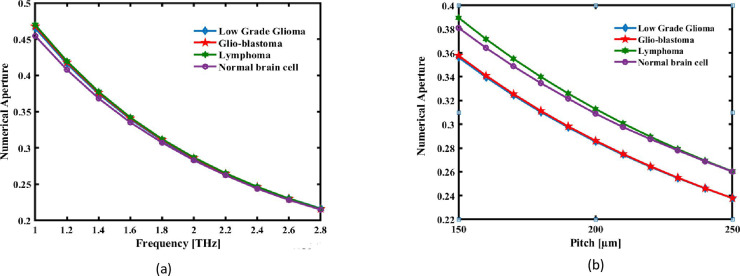
The NA of the recommended the detection is shown as an expression of (a) pitch at 2.2 THz and (b) signal frequency at a set pitch of 220 µm.

Fig 6(a) and 6(b) illustrate the relationship between Numerical Aperture and pitch and frequency variations, respectively. Most of the decrease in numerical aperture with an increase in frequency and pitch is due to changes in light confinement and refractive index contrast between core and cladding. At higher frequencies of the terahertz spectrum, the wavelength of the electromagnetic waves becomes shorter, hence being better localized within the core. With an increase in the pitch P that defines the distance between air holes in the cladding, the effective refractive index contrast between the core and the cladding becomes smaller. The acceptance angle of the fiber is reduced, hence lowering the NA. The air-hole structure in the cladding is designed to be highly symmetrical, further reducing the divergence of guided light, which enhances light confinement but restricts the angular range of light that can enter the fiber. As a result, the numerical aperture decreases, which aligns with the sensor design goal of improving precision in refractive index detection for brain lesion analysis while maintaining efficient light guidance.

In both situations, as pitch and frequency rise, the NA falls, as the data clearly demonstrate. At an optimum pitch of 220micrometres and 2.2 THz in frequency, the NA values for the Low Grade Glioma, Glio-blastoma, Lymphoma and Normal brain cell are, respectively 0.2641, 0.2646, 0.2651 and 0.2622. The photonic crystal fiber (PCF) numerical aperture (NA) controls the light collection efficiency, which in turn impacts the sensor’s sensitivity and resolution. A higher NA improves the capacity of the detector to identify even slight shifts in refractive index (RI) and captures a wider variety of light angles.

**Fig 7:**
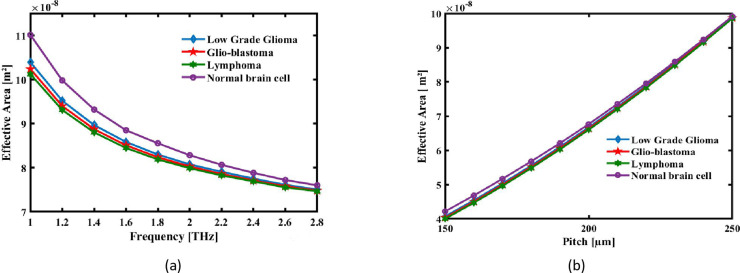
The effective area of the recommended the detection is shown as an expression of (a) pitch at 2.2 THz and (b) signal frequency at a set pitch of 220 µm.

The dispersed light-emitting power is spread across the area of the fiber cross-sectional surface, which is measured by Effective Area. Total internal reflection and photonic bandgap processes can work together to restrict light in PCFs because of their unique microstructure. PCFs may have a useful space that is significantly lower than that of traditional fibres due to their confinement, which makes them desirable for a range of applications. It is possible to identify the effective area using the formula that follows [68]:


$$A_{eff}=\frac{{\left[\int I\left(r\right)rdr\right]}^{2}}{{\left[\int I^{2}\left(r\right)rdr\right]}^{2}}$$ (6)

where *I(r)=|E | *^*2*^ is the dispersion of the sensing analyte’s electric field. This PCF’s EA at different frequencies of operation and pitches appears in Fig 7(a) and 7(b) respectively. It should be mentioned that with the reduction in wavelength, the optical field becomes more localized within the fiber core and the effective area reduces. This, in fact enhances the light-matter interaction, which may be desired in applications requiring high sensitivity. The opposite happens with the increase in pitch, P, which is the spacing between air holes in the photonic crystal cladding-an increase in effective area. This spreads out the optical field over a much larger area by reducing the confining nature that is introduced due to the finite pitch value. Consequently, the growth of an effective area as the pitch is enlarged implies an improved guided mode-analyte overlap sensitivity for the refractive index sensor in brain lesion detection. The frequency-pitch balance has to be achieved to ensure the performance of the sensor, enabling precise and reliable detection of brain lesions.The figure illustrates how the signal with greater frequency is restricted to the core and how the effective region shrinks with increasing operating frequency. The useful region of the optical detector at 2.2 THz and ideal pitch for the Low Grade Glioma, Glio-blastoma, Lymphoma and Normal brain cell are, respectively, 7.9083 × 10^-08^ m^2^, 7.8578 × 10^-08^ m^2^, 7.8270 × 10^-08^ m^2^, and 8.06m68 × 10^-08^ m^2^.

In PCF, the determination of the light phase that travels through the fibre is indicated to as “spot size”. The elements that impact PCF spot size include the light spectrum, and the manner of operation, and fibre architecture.

Finally, An important factor for sensing applications is the recommended spot size of the sensor, which is investigated. For sensing applications, large spot sizes are preferred, and they can be calculated with the following formula [69]:


$$W_{eff}=R\times \left(.65\times 1.619\times V^{-1.5}+2.789\times V^{-6}\right)$$ (7)

**Fig 8:**
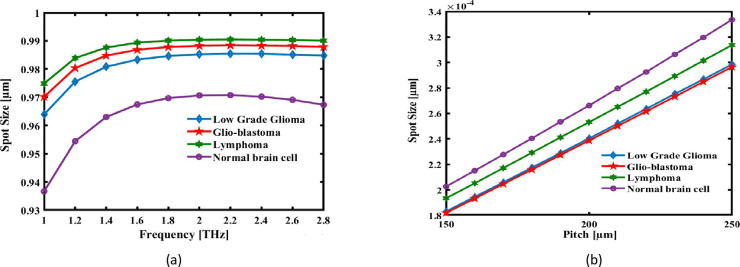
The spot size of the recommended the detection is shown as an expression of (a) pitch at 2.2 THz and (b) signal frequency at a set pitch of 220 µm.

Where, *V* is the definition of the normalised frequency value and *R* is the definition of the hexagonal core’s radius. As Fig 8(a) illustrates, Location of the detecting fibre size decreases with increasing frequency. The spot size decreases with frequency increase because the electromagnetic field is better confined for higher frequencies. While the wavelength becomes shorter, the optical field becomes more concentrated within the core, reducing the spread of the beam and therefore the spot size, which indeed is desirable when higher resolution and sensitivity are required in sensing applications. Meanwhile, the pitch of the air holes in the photonic crystal cladding, shown by the letter P, increases the spot size. Larger values of the pitch of the structure make the mode less tightly confined to the core region and therefore more distributed. Because the larger spot size is permitted to interact more with the surrounding analyte, this increases the capability of the sensor by way of detecting changes in refractive index. The tradeoff between frequency and pitch is important for optimization in the performance of the sensor, enabling it to detect brain lesions with high sensitivity and resolution. Pitch parameter increases also cause a rise in the sensor’s spot size as Fig 8(b). The spot size of the sensor fibre at 2.2 THz, at optimal pitch for the Low Grade Glioma, Glio-blastoma, Lymphoma and Normal brain cell are, respectively, 2.6358 × 10^-04^µm, 2.6157 × 10^-04^ µm, 2.6015 × 10^-04^ µm, and 2.7279 × 10^-04^ µm.

The terahertz spectrum-based refractive index detection sensor PCF was developed using Zeonex due to its excellent optical properties: high transparency, low absorption loss, biocompatibility, and robust tensile strength. It is suitable for biological sensing applications, providing a precise detection of refractive index variation in brain lesions. It improves the light confinement and potential for sensing with the round core featuring flower-petal-shaped air channels, while simplifying fabrication based on one constant (P) that defines the features of core and cladding. Perfect matching to reduce back-reflection increases the efficiency of the sensor by absorbing the leakage radiation with an added layer. It has a mesh structure with carefully designed air holes combined with 5971 elements for the best guidance of the beams with transparency; hence, an external connection was provided for the tailored dispersion and noise properties. It provides an extremely sensitive and low-cost sensor for refractive index values for change, correctly identifying brain lesions, thus it’s a new versatile tool for medical diagnostics.

The proposed PCF sensor outperforms existing tumor-detection technologies in accuracy and response time. Using Zeonex as the base material ensures high transparency, low optical loss, and biocompatibility, while the circular core and air-channel cladding enhance light confinement and sensitivity. Perfectly matched layers reduce back-reflection, increasing efficiency and enabling rapid responses. Fabrication is simplified with a single constant for core and cladding dimensions, making the process cost-effective and scalable. The sensor’s high sensitivity to refractive index changes offers reliable detection of brain lesions like glioblastoma and lymphoma, demonstrating its versatility and superiority over traditional methods which is compared in Table 1.

**Table 1:** Comparison of our suggested sensor with the previously published sensor.

References	Structure of the Photonic Crystal Fiber	Analyte	Frequency (THz)	Sensitivity (%)	Confinement Loss (dB/m)	Effective Material Loss (cm^-1^)
**PCF** [70]	Heptagonal cladding surrounding a round core	adulteration of petrol with kerosene	*f* = 2.8 THz	96.87%	7.21 × 10^ − 4^	0.0072
**PCF** [71]	Heptagonal cladding in 5 layers with round air holes	Ethanol Benzene Water	*f* = 1.0 THz	68.48%, 69.20%, 66.78%	2.13 × 10^-9^ 1.92 × 10^-9^ 2.70 × 10^-9^	– – –
**PCF** [72]	Decagonal solid core PCF	glucose plasma WBC RBC	*f* = 1.0 THz	84.55%, 85.09%, 85.62% 87.68%	7.92 × 10-^09^ 6.66 × 10^–09^ 3.14 × 10^-09^ 1.86 × 10^–09^	– – – –
**PCF** [73]	Eight symmetrical air holes with octagonal core	Cholesterol (LDL)	*f* = 2.2 THz	98.75%	3.14 × 10^ − 20^	0.0008
**PCF** [74]	Rectangular core and air hole	Breast cancer	*f* = 2.8 THz	92.2%	6.52 × 10^ − 14^	0.0117
**PCF** [75]	mono-rectangular holes with elliptical cladding	Cancer cell Normal cell	*f* = 2.1 THz	81.38% 65.83%	5.828 × 10^ − 25^ 3.072 × 10^ − 27^	– –
**PCF** [76]	A radial hole drilled into the core is round.	White matter Cerebrospinal fluid Gray matter Multi-sclerosis Oligodendroglioma Wall of solid brain Medulloblastoma Low grade glioma Metastasis Lymphoma Glioblastoma	*f* = 2.2 THz	94.7% 89.0% 93.7% 89.8% 90.8% 89.7% 96.0% 95.6% 97.5% 96.7% 96.7%	1.24 × 10^ − 10^ 8.19 × 10^ − 9^ 3.04 × 10^ − 10^ 4.97 × 10^ − 9^ 2.80 × 10^ − 9^ 5.33 × 10^ − 9^ 2.80 × 10^ − 11^ 4.47 × 10^ − 11^ 2.21 × 10^ − 11^ 2.88 × 10^ − 11^ 1.76 × 10^ − 11^	0.008983 0.01497 0.009983 0.014066 0.013102 0.01419 0.007544 0.007965 0.005942 0.006802 0.007292
**This PCF**	Ten symmetrical air holes with circular core	Low Grade Glioma, Glio-blastoma, Lymphoma Normal brain cell	*f* = 2.2 THz	98.54% 98.84% 99.04% 97.07%	1.32 × 10^ − 13^ 6.31 × 10^ − 15^ 1.07 × 10^ − 13^ 1.54 × 10^ − 13^	0.003441 0.003013 0.002708 0.005281

Manufacturing of PCFs via 3D printing involves designing such a complicated structure of the fiber using 3D modeling software, where both the core and cladding features of the fiber are exactly defined. Translate this design into a 3D printer that may deal with materials like resin or photopolymer [77,78]. The refractive index sensor for brain tumor detection faces limitations related to material and structural complications. While Zeonex offers high transparency and biocompatibility, it lacks flexibility and presents manufacturing challenges compared to alternatives like PMMA or silica. The complex design requires precise fabrication, increasing production costs and complexity. Additionally, material quality variations and environmental factors may impact performance, necessitating careful alignment and calibration for clinical use. These challenges highlight the need for further developments to ensure reliable, large-scale applications of this sensor technology. The engineered air channels and Perfectly Matched Layer reduce back-reflection, enhancing the sensor’s efficiency and sensitivity to refractive index variations in brain lesions like glioblastoma and lymphoma. Its simple design minimizes manufacturing costs while maintaining high performance for fast, accurate diagnostics. This enables real-time identification of brain abnormalities, improving patient outcomes in critical situations. To enhance the PCF sensor for broader applications, material improvements can address Zeonex’s rigidity and production challenges by using flexible options like PMMA or silica. Multilayered coatings and adaptive air-hole designs could improve sensitivity and light confinement for diverse tissue types. Adding a protective outer layer would boost reliability in harsh environments, while incorporating tunable terahertz frequencies expands detection capabilities. Refining simulation techniques, such as mesh optimization, ensures high accuracy, extending the sensor’s use in medical diagnostics, security, and environmental monitoring.

## 4. Conclusion

The development of a PCF sensor capable of identifying hazardous components was our main objective. Our very capable sensing device that we have proposed was created in response to this circumstance. The recommended detectors’ maximum relative sensitivities (Max RS) at f =  2.0 THz are 98.54% for low-grade glioma, 98.84% for glioblastoma, 99.049% for lymphoma, and 97.075% for normal brain cells. COMSOL MULTIPHYSICS designing and analyzing the expected PCF sensor was done efficiently with the use of simulation software. Air channels shaped like spiders make up the special covering area of the scanner. A hex center is located midway through this Photonic Crystal Fiber. Through its ability to sense even minute fluctuations in RI, our planned sensor facilitates the identification of tumor at low concentrations. Because of its rapid reflexes, it can monitor the quantities of Low Grade Glioma, Glio-blastoma, Lymphoma and Normal brain cell instantly, which is essential for moving forward quickly in hazardous conditions.. Because of their high sensitivity to changes in refractive index between normal and tumorous tissues, the recently developed PCF sensors which are based on the terahertz spectrum are adaptable and trustworthy for identifying brain tumors. Low dispersion, improved detection precision, and accurate light guidance are guaranteed by the photonic crystal fiber design. These detectors are an exciting tool for medical imaging and tumor monitoring since they may be used for a variety of diagnostic purposes and offer non-invasive, accurate, and efficient detection of brain lesions. The PCF sensor faces challenges in clinical and hazardous applications, including limited material flexibility and complex manufacturing compared to traditional materials like PMMA or silica. Its intricate design requires precise fabrication, complicating mass production. Environmental factors, such as temperature and mechanical stress, may affect performance due to terahertz spectrum sensitivity. Advanced fabrication methods like 3D printing, protective coatings, and robust housing can mitigate these issues, ensuring durability and accuracy. Proper training of clinicians and operators will enhance the sensor’s successful implementation across various settings.

## References

[1] Yadav CS, Upadhyay A, Singh V. Sensing performance of Bragg fibers having a defect layer utilizing confinement loss analysis for brain cancer cells detection. Opt Commun. 2023;526:128887. doi: 10.1016/j.optcom.2022.128887

[2] Zheng S, Shan B, Ghandehari M, Ou J. Sensitivity characterization of cladding modes in long-period gratings photonic crystal fiber for structural health monitoring. Measurement. 2015;72:43–51. doi: 10.1016/j.measurement.2015.04.014

[3] Zheng S, Zhu Y, Krishnaswamy S. Fiber humidity sensors with high sensitivity and selectivity based on interior nanofilm-coated photonic crystal fiber long-period gratings. Sensors Actuat B: Chem. 2013;176:264–74. doi: 10.1016/j.snb.2012.09.098

[4] Rodrigues Pinto AM, Baptista JM, Santos JL, Lopez-Amo M, Frazão O. Micro-displacement sensor based on a hollow-core photonic crystal fiber. Sensors (Basel). 2012;12(12):17497–503. doi: 10.3390/s121217497 23247414 PMC3571850

[5] Ademgil H, Haxha S. Endlessly single mode photonic crystal fiber with improved effective mode area. Opt Commun. 2012;285(6):1514–8. doi: 10.1016/j.optcom.2011.10.067

[6] Saad NM, El-Rabaie E-SM, Khalaf AAM. Study of the optical properties for low-loss rectangular porous core photonic crystal fiber (R-PCF) topology for biomedical sensing application. J Opt. 2023;53(3):2481–502. doi: 10.1007/s12596-023-01464-8

[7] Anas MT, Asaduzzaman S, Ahmed K, Bhuiyan T. Investigation of highly birefringent and highly nonlinear Hexa Sectored PCF with low confinement loss. Results Phys. 2018;11:1039–43. doi: 10.1016/j.rinp.2018.11.013

[8] Kabir S, Razzak SMA. An enhanced effective mode area fluorine doped octagonal photonic crystal fiber with extremely low loss. Photonics Nanostructures - Fundam Appl. 2018;30:1–6. doi: 10.1016/j.photonics.2018.02.002

[9] Maji PS, Chaudhuri PR. Dispersion properties of the square-lattice elliptical-core PCFs. Am J Opt Photonics. 2014.

[10] Rahman M, Akhter N, Rahat Ali SK. Comparative study of different fiber materials based PCF for enhancing medical imaging quality. Results Opt. 2023;13:100578. doi: 10.1016/j.rio.2023.100578

[11] Kaur V, Singh S. Extremely sensitive multiple sensing ring PCF sensor for lower indexed chemical detection. Sens Bio-Sensing Res. 2017;15:12–6. doi: 10.1016/j.sbsr.2017.05.001

[12] Almewafy BH, Areed NFF, Hameed MFO, Obayya SSA. Modified D-shaped SPR PCF polarization filter at telecommunication wavelengths. Opt Quant Electron. 2019;51(6):193. doi: 10.1007/s11082-019-1885-x

[13] Markin AV, Markina NE, Goryacheva IYu. Raman spectroscopy based analysis inside photonic-crystal fibers. TrAC Trends Anal Chem. 2017;88:185–97. doi: 10.1016/j.trac.2017.01.003

[14] Mou FA, Rahman MdM, Islam MR, Bhuiyan MIH. Development of a photonic crystal fiber for THz wave guidance and environmental pollutants detection. Sens Bio-Sensing Res. 2020;29:100346. doi: 10.1016/j.sbsr.2020.100346

[15] Pandey P, Yadav S, Dwivedi DK, Lohia P, Mishra AC, Yadav RK, et al. Performance analysis of highly sensitive PCF sensor for drug detection. J Opt. 2024. doi: 10.1007/s12596-024-01865-3

[16] Shakya A, Cheng F, Carmon T. Ultracoherent emission by orthogonal lasers. Integrated Photonics Platforms III, SPIE; 2024 Jun. p. 79–81. doi: 10.1117/12.3022322

[17] Shakya AK, Singh S. Performance analysis of a developed optical sensing setup based on the beer-lambert law. Plasmonics. 2023;19(1):447–55. doi: 10.1007/s11468-023-01979-7

[18] Kumar Shakya A, Singh S. Design of novel Penta core PCF SPR RI sensor based on fusion of IMD and EMD techniques for analysis of water and transformer oil. Measurement. 2022;188:110513. doi: 10.1016/j.measurement.2021.110513

[19] Shakya AK, Singh S. Designing of a novel PCF biosensor having octagonal core and based on SPR for chemical and heavy metal sensing. 2022 12th International Conference on Cloud Computing, Data Science & Engineering (Confluence); 2022 Jan. p. 171–5. doi: 10.1109/confluence52989.2022.9734120

[20] Shakya AK, Singh S. State of the art alliance of refractive index sensing and spectroscopy techniques for household oils analysis. Plasmonics. 2023;18(6):2347–64. doi: 10.1007/s11468-023-01940-8

[21] Shakya AK, Singh S. Development of a generalized Fourier transform model for distinct household oil samples by performing spectroscopy analysis. Results Opt. 2023;10:100355. doi: 10.1016/j.rio.2023.100355

[22] Ghodrati M, Uniyal A. Exploring metasurface-based biosensor: new frontiers in sensitivity and versatility for biomedical applications. Plasmonics. 2024. doi: 10.1007/s11468-024-02640-7

[23] Uniyal A, Kumba K, Dhiman G, Ahmed MZ, Pal A, Pal D, et al. Advanced SPR sensor for human sperm analysis: leveraging silver and nanomaterials for enhanced performance. Plasmonics. 2024. doi: 10.1007/s11468-024-02606-9

[24] Pal A, Trabelsi Y, Sarkar P, Yadav RB, Sharma M, Uniyal A, et al. Plasmonic pregnancy detector: enhancing sensitivity with SPR sensor. Opt Quant Electron. 2024;56(9):1404. doi: 10.1007/s11082-024-07342-2

[25] Pal A, Trabelsi Y, Sarkar P, Sharma M, Kumar M, Uniyal A. Tuning sensitivity of surface plasmon resonance sensor based on ZnO layer and CaF2 prism for the recognition of SARS-CoV-2. J Mater Sci: Mater Electron. 2024;35(22):1523. doi: 10.1007/s10854-024-13287-9

[26] Zaman MU, Pal A, Uniyal A, Alqhtani NR, Sharma M, Farzan MSA, et al. ZnO and antimonene-based surface plasmon resonance sensor for enamel, dentin, and cementum layer detection in human teeth. J Mater Sci: Mater Electron. 2024;35(27):1831. doi: 10.1007/s10854-024-13471-x

[27] A survey on terahertz communications. [cited 2024 Sep 17]. [Online]. Available from: https://ieeexplore.ieee.org/abstract/document/8663550

[28] Elhelw AR, Ibrahim MSS, Rashed ANZ, Mohamed AE-NA, Hameed MFO, Obayya SSA. Highly sensitive bilirubin biosensor based on photonic crystal fiber in terahertz region. Photonics. 2023;10(1):68. doi: 10.3390/photonics10010068

[29] Single-mode mid-IR guidance in a hollow-core photonic crystal fiber. [cited 2024 Sep 17]. [Online]. Available from: https://opg.optica.org/oe/fulltext.cfm?uri=oe-13-18-7139&id=85389 10.1364/opex.13.007139 19498737

[30] Jibon RH, Bulbul AA-M, Rahaman MdE. Numerical investigation of the optical properties for multiple PCF structures in the THz regime. Sens Bio-Sensing Res. 2021;32:100405. doi: 10.1016/j.sbsr.2021.100405

[31] Yadav S, Lohia P, Dwivedi DK. Quantitative analysis of highly efficient PCF-based sensor for early detection of breast cancer cells in THz regime. J Opt. 2023;53(3):2642–55. doi: 10.1007/s12596-023-01404-6

[32] Jibon RH, Ahmed M, Hasan MdK. Identification of detrimental chemicals of plastic products using PCF in the THz regime. Meas: Sens. 2021;17:100056. doi: 10.1016/j.measen.2021.100056

[33] Maidi AM, Salam R, Kalam MdA, Begum F. Design and simulation of photonic crystal fibre sensor for harmful chemicals detection in polycarbonate plastics. Opt Quant Electron. 2023;56(1):31. doi: 10.1007/s11082-023-05601-2

[34] Nakaema WM, Hao Z-Q, Rohwetter P, Wöste L, Stelmaszczyk K. PCF-based cavity enhanced spectroscopic sensors for simultaneous multicomponent trace gas analysis. Sensors (Basel). 2011;11(2):1620–40. doi: 10.3390/s110201620 22319372 PMC3274003

[35] High extinction ratio and large bandwidth PCF polarization filter with gold-wires coated by monocrystalline silicon. [cited 2024 Sep 17]. [Online]. Available from: https://ieeexplore.ieee.org/abstract/document/9839593

[36] Ferdous AHMI, Rani L, Islam MS, Noor KS, Roy S, Eid MMA, et al. Development and enhancement of PCF-based sensors for terahertz-frequency region breast cancer cell detection. Cell Biochem Biophys. 2024;82(3):2837–52. doi: 10.1007/s12013-024-01399-2 38982022

[37] Luo W, Zou D, Liu T, Gao S. A fiber optic laser-ultrasonic transmitter based on collapsed photonic crystal fiber for ultrasonic testing. Struct Health Monit. 2024:14759217241267821. doi: 10.1177/14759217241267821

[38] Islam N, Masum MdMU, Arif MdFH, Asaduzzaman S, Roy M, Yousuf MA. Enhanced sensitivity of open channel SPR-based PCF sensor employing plasmonic materials for analyte sensing. Plasmonics. 2022;17(5):2075–87. doi: 10.1007/s11468-022-01691-y

[39] Zhan X, Liu Y, Chen Z, Luo J, Yang S, Yang X. Revolutionary approaches for cancer diagnosis by terahertz-based spectroscopy and imaging. Talanta. 2023;259:124483. doi: 10.1016/j.talanta.2023.124483 37019007

[40] Yamaguchi S, Fukushi Y, Kubota O, Itsuji T, Ouchi T, Yamamoto S. Brain tumor imaging of rat fresh tissue using terahertz spectroscopy. Sci Rep. 2016;6:30124. doi: 10.1038/srep30124 27456312 PMC4960480

[41] El-Shenawee M, Vohra N, Bowman T, Bailey K. Cancer detection in excised breast tumors using terahertz imaging and spectroscopy. Biomed Spectrosc Imaging. 2019;8(1–2):1–9. doi: 10.3233/bsi-190187 32566474 PMC7304303

[42] Shi J, Wang Y, Chen T, Xu D, Zhao H, Chen L, et al. Automatic evaluation of traumatic brain injury based on terahertz imaging with machine learning. Opt Express. 2018;26(5):6371–81. doi: 10.1364/OE.26.006371 29529829

[43] Abuawad M, Daqour A, Alkaiyat A, Rjoub A, Zahra WA, Issa N, et al. Epidemiology of primary brain tumor among adolescents and adults in Palestine: a retrospective study from 2018 to 2023. BMC Neurol. 2024;24(1):168. doi: 10.1186/s12883-024-03677-1 38783212 PMC11112926

[44] Ostrom QT, Price M, Neff C, Cioffi G, Waite KA, Kruchko C, et al. CBTRUS statistical report: primary brain and other central nervous system tumors diagnosed in the United States in 2015-2019. Neuro Oncol. 2022;24(Suppl 5):v1–95. doi: 10.1093/neuonc/noac202 36196752 PMC9533228

[45] Batool A, Byun Y-C. Brain tumor detection with integrating traditional and computational intelligence approaches across diverse imaging modalities - Challenges and future directions. Comput Biol Med. 2024;175:108412. doi: 10.1016/j.compbiomed.2024.108412 38691914

[46] Kabir TF, Kunos CA, Villano JL, Chauhan A. Immunotherapy for medulloblastoma: current perspectives. Immunotargets Ther. 2020;9:57–77. doi: 10.2147/ITT.S198162 32368525 PMC7182450

[47] Ostrom QT, Price M, Ryan K, Edelson J, Neff C, Cioffi G, et al. CBTRUS statistical report: pediatric brain tumor foundation childhood and adolescent primary brain and other central nervous system tumors diagnosed in the United States in 2014-2018. Neuro Oncol. 2022;24(Suppl 3):iii1–38. doi: 10.1093/neuonc/noac161 36066969 PMC9447434

[48] Mohammed NA, Khedr OE, El-Rabaie E-SM, Khalaf AAM. Brain tumors biomedical sensor with high-quality factor and ultra-compact size based on nanocavity 2D photonic crystal. Alex Eng J. 2023;64:527–40. doi: 10.1016/j.aej.2022.09.020

[49] Bin Murshed Leon MJ, Disha AS. A simple structure of PCF based sensor for sensing sulfur dioxide gas with high sensitivity and better birefringence. Sens Int. 2021;2:100115. doi: 10.1016/j.sintl.2021.100115

[50] Haque E, Mahmuda S, Hossain MdA, Hai NH, Namihira Y, Ahmed F. Highly sensitive dual-core PCF based plasmonic refractive index sensor for low refractive index detection. IEEE Photonics J. 2019;11(5):1–9. doi: 10.1109/jphot.2019.2931713

[51] Bulbul AA-M, Kouzani AZ, Mahmud MAP, Nahid A-A. Design and numerical analysis of a novel rectangular PCF (R-PCF)-based biochemical sensor (BCS) in the THz regime. Int J Opt. 2021;2021:1–16. doi: 10.1155/2021/5527724

[52] Mohammed AZ. Photonic crystal fiber Mach-Zehnder interferometer pH sensing. AIP Conf Proc. 2018;2045:020010. doi: 10.1063/1.5080823

[53] Dong X, Luo Z, Du H, Sun X, Yin K, Duan J. Highly sensitive strain sensor based on a novel Mach–Zehnder mode interferometer with TCF-PCF-TCF structure. Opt Lasers Eng. 2019;116:26–31. doi: 10.1016/j.optlaseng.2018.12.007

[54] Lo SM, Hu S, Gaur G, Kostoulas Y, Weiss SM, Fauchet PM. Photonic crystal microring resonator for label-free biosensing. Opt Express. 2017;25(6):7046–54. doi: 10.1364/OE.25.007046 28381045

[55] Nouman WM, Abd El-Ghany SE-S, Sallam SM, Dawood A-FB, Aly AH. Biophotonic sensor for rapid detection of brain lesions using 1D photonic crystal. Opt Quant Electron. 2020;52(6):287. doi: 10.1007/s11082-020-02409-2

[56] Sun J, Lee SJ, Wu L, Sarntinoranont M, Xie H. Refractive index measurement of acute rat brain tissue slices using optical coherence tomography. Opt Express. 2012;20(2):1084–95. doi: 10.1364/OE.20.001084 22274454 PMC3501791

[57] Karki B, Sarkar P, Mahmoud KH, Alsubaie ASA, Sharma M. Detection of organic material using tungsten ditelluride based surface plasmon resonance sensor. Plasmonics. 2024. doi: 10.1007/s11468-024-02356-8

[58] Karki B, Alsubaie AS, Sarkar P, Sharma M, Ali NB. Detection of skin, cervical, and breast cancer using au–ag alloy and WS2-based surface plasmon resonance sensor. Plasmonics. 2024. doi: 10.1007/s11468-024-02521-z

[59] Karki B, Uniyal A, Pal A, Srivastava V. Advances in surface plasmon resonance-based biosensor technologies for cancer cell detection. Int J Opt. 2022;2022:1–10. doi: 10.1155/2022/1476254

[60] Karki B, Pal A, Alsubaie AS, Mahmoud KH, Sharma M. Detection of pathogens in water using long range surface plasmon resonance biosensor: numerical investigation. Physica B: Condens Matter. 2024;695:416503. doi: 10.1016/j.physb.2024.416503

[61] Karki B, Pal A, Sarkar P, Yadav RB, Muduli A, Trabelsi Y. ZnO-silicon enhanced surface plasmon resonance sensor for chemical sensing. Silicon. 2024;16(9):3861–72. doi: 10.1007/s12633-024-02973-2

[62] Karki B, Uniyal A, Sharma M, Yadav RB, Buduma P. Tuning sensitivity of bimetallic, MXene and graphene-based SPR biosensors for rapid malaria detection: a numerical approach. J Comput Electron. 2024;23(4):920–9. doi: 10.1007/s10825-024-02191-4

[63] Jibon RH, Khodaei A, Priya PP, Rashed ANZ, Ahammad SH, Hossain MdA. ZEONEX based hollow rectangular core photonic crystal fiber (PCF) sensor design and numerical investigation for alcohol detection of variant classes. Opt Quant Electron. 2023;55(11):977. doi: 10.1007/s11082-023-05267-w

[64] S SB, Bahaddur I. Rapid detection of brain lesions using biosensor based 2D photonic crystal. 2021 IEEE International Conference on Electronics, Computing and Communication Technologies (CONECCT). Bangalore, India: IEEE; 2021 Jul. p. 1–6. doi: 10.1109/conecct52877.2021.9622564

[65] Brechet F, Marcou J, Pagnoux D, Roy P. Complete analysis of the characteristics of propagation into photonic crystal fibers, by the finite element method. Opt Fiber Technol. 2000;6(2):181–91. doi: 10.1006/ofte.1999.0320

[66] Kumar Paul B, Haque MdA, Ahmed K, Sen S. A novel hexahedron photonic crystal fiber in terahertz propagation: design and analysis. Photonics. 2019;6(1):32. doi: 10.3390/photonics6010032

[67] Ferdous AHMI, Naim MNR, Noor KS, Kundu D, Rashed ANZ. Innovative refractive index sensor utilizing terahertz spectrum for early cancer detection: a photonic crystal fiber approach. Cell Biochem Biophys. 2025;83(1):489–505. doi: 10.1007/s12013-024-01479-3 39127860

[68] Noor KS, Iftekharul Ferdous AHM, Bani MostM, Sadeque MdG. Floral-core terahertz photonic crystal fiber bio-sensor: an exclusive approach to recognizing milk from various animal species. Appl Food Res. 2024;4(2):100481. doi: 10.1016/j.afres.2024.100481

[69] Kundu D, Faruk MdO, Khandakar K, Ferdous AHMI, Bani MostM, Noor KS. Terahertz photonic crystal fiber sensor: Evaluating performance for chemical identification. Int Commun Heat Mass Transf. 2024;156:107615. doi: 10.1016/j.icheatmasstransfer.2024.107615

[70] Ferdous AHMI, Anower MdS, Musha A, Habib MdA, Shobug MdA. A heptagonal PCF-based oil sensor to detect fuel adulteration using terahertz spectrum. Sens Bio-Sensing Res. 2022;36:100485. doi: 10.1016/j.sbsr.2022.100485

[71] Hossain MdS, Sen S. Design and performance improvement of optical chemical sensor based photonic crystal fiber (PCF) in the terahertz (THz) wave propagation. Silicon. 2020;13(11):3879–87. doi: 10.1007/s12633-020-00696-8

[72] Kumar A, Verma P, Jindal P. Decagonal solid core PCF based refractive index sensor for blood cells detection in terahertz regime. Opt Quant Electron. 2021;53(4):165. doi: 10.1007/s11082-021-02818-x

[73] Rahman MdM, Mou FA, Bhuiyan MIH, Islam MR. Photonic crystal fiber based terahertz sensor for cholesterol detection in human blood and liquid foodstuffs. Sens Bio-Sensing Res. 2020;29:100356. doi: 10.1016/j.sbsr.2020.100356

[74] Bulbul AA-M, Rahaman H, Biswas S, Hossain MB, Nahid A-A. Design and numerical analysis of a PCF-based bio-sensor for breast cancer cell detection in the THz regime. Sens Bio-Sensing Res. 2020;30:100388. doi: 10.1016/j.sbsr.2020.100388

[75] Yadav S, Lohia P, Dwivedi DK. Eminently sensitive mono-rectangular photonic crystal fiber-based sensor for cancer cell detection in THz regime. J Opt. 2023;53(1):528–37. doi: 10.1007/s12596-023-01191-0

[76] Mohammed NA, Khedr OE, El-Rabaie E-SM, Khalaf AAM. Early detection of brain cancers biomedical sensor with low losses and high sensitivity in the terahertz regime based on photonic crystal fiber technology. Opt Quant Electron. 2023;55(3):230. doi: 10.1007/s11082-022-04515-9

[77] Luo Y, Chu Y, Wang J, Fu X, Canning J, Cao Y, et al. All solid photonic crystal fiber enabled by 3D printing fiber technology for sensing of multiple parameters. Adv Sens Res. 2024;3(11):2300205. doi: 10.1002/adsr.202300205

[78] Bertoncini A, Liberale C. 3D printed waveguides based on photonic crystal fiber designs for complex fiber-end photonic devices. Optica. 2020;7(11):1487. doi: 10.1364/optica.397281

